# Spontaneus bilateral pedicle fracture 30 years after Harrington Instrumentation for idiopathic scoliosis: a case report

**DOI:** 10.1186/1752-1947-6-29

**Published:** 2012-01-23

**Authors:** Peter Obid, Alexander Richter, Hüseyin Übeyli, Thomas Niemeyer

**Affiliations:** 1 Asklepios Klinik St. Georg, Abt. für Wirbelsäulen- und Skoliosechirurgie, Lohmühlenstraße 5, 20099 Hamburg, Germany

## Abstract

**Introduction:**

Spontaneous fractures of the spine are a common entity. They usually occur in older people with osteoporosis. This case is presented on account of its rarity. To the best of the authors' knowledge only one case of an osteoporotic pedicle fracture after Harrington Instrumentation has been described before.

**Case presentation:**

We report the case of a 46-year-old Caucasian woman who underwent surgery due to idiopathic scoliosis with a Harrington Instrumentation (T4 to L3) 30 years ago. During the operation she was infected with hepatitis C while receiving erythrocyte concentrates and has suffered from liver cirrhosis since then. She presented with a sudden pain in her lower back and paraesthesia in both her legs but no other neurological symptoms. A computed tomography scan showed a bilateral pedicle fracture of L3 and an additional compression fracture of L4. In the first session we performed a dorsal stabilization with massive intraoperative bleeding and a postoperative failure of liver synthesis. In a second session an additional ventral augmentation was done. After the second operation she developed a hepatorenal syndrome. Both operations left the patient in a very critical state which led to a prolonged stay in the intensive care and rehabilitation unit. At her 12-month follow-up visit, she was free of complaints.

**Conclusion:**

The un-physiological load of the spine after Harrington Instrumentation can lead to osteoporosis due to inactivity even in younger patients. Although these implants are not used anymore one should keep this possibility in mind when dealing with patients who have received Harrington rods in surgical procedures.

## Introduction

The Harrington rods were the first implants which were successfully used for the surgical correction of scoliosis [1]. Until pedicle screws were available the Harrington rods had been the gold standard in scoliosis surgery but they were not able to correct the sagittal profile or the vertebral rotation. With the pedicle-screw systems used today a three-dimensional correction of the deformity is possible [2]. However, some problems remain. Every instrumentation of the spine leads to decreased volumetric densitiy of the bone which makes further degeneration more likely [3]. In this case the patient's advanced liver cirrhosis led to an additional hepatic osteopenia [4,5]. Choudhary *et al*. showed that patients with liver cirrhosis have a higher risk of developing an osteodystrophy [6]. We present the case of a 46-year-old woman with a Harrington instrumentation and liver cirrhosis who incurred a spinal fracture without a trauma.

## Case presentation

**Figure 1:**
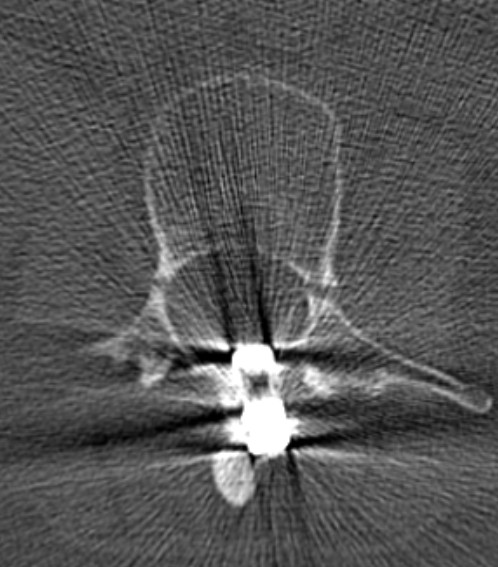
A computed tomography scan shows the fractured pedicles of L3

**Figure 2:**
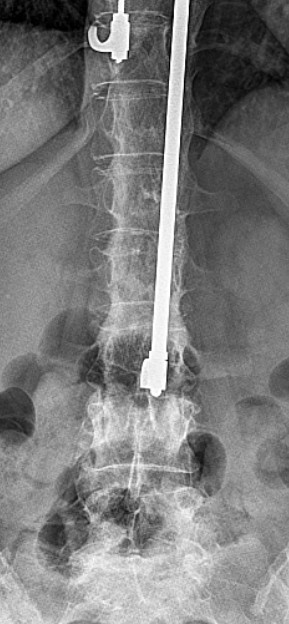
X-ray of the lumbar spine in anterior-posterior projection

**Figure 3:**
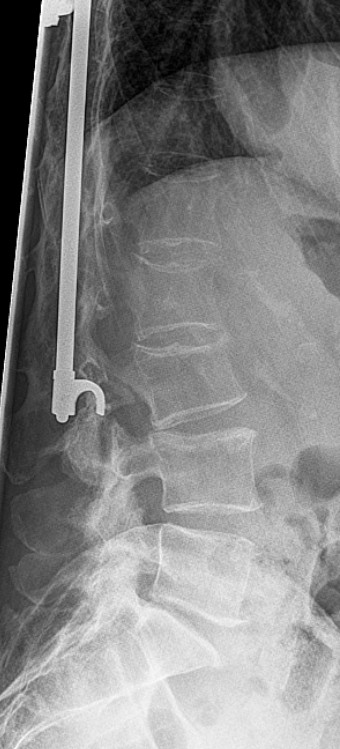
X-ray of the lumbar spine in lateral projection showing the fractured pedicle

**Figure 4:**
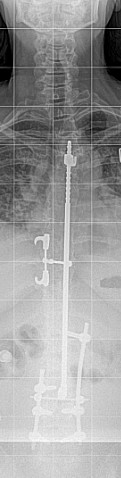
X-ray of the spine in anterior-ppsterior projection after dorsal spondylodesis

**Figure 5:**
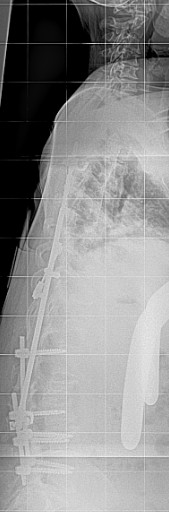
X-ray of the spine in lateral projection after dorsal spondylodesis showing the progressive thoracolumbar kyphosis and looming cutting-out of the pedicle screw in L1

**Figure 6:**
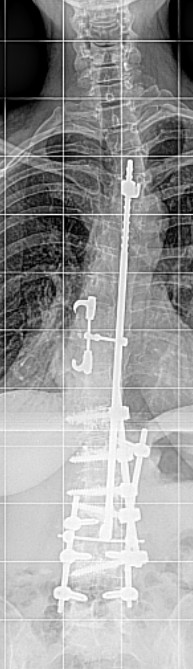
X-ray of the spine in anterior-posterior projection one year after additional ventral augmentation

**Figure 7:**
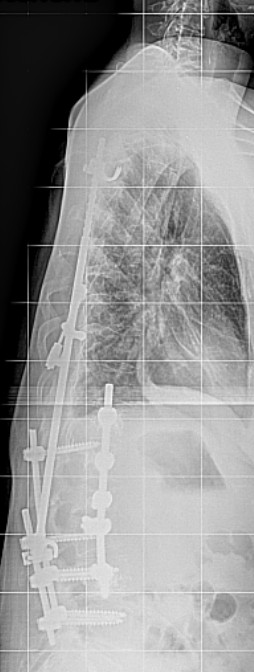
X-ray of the spine in lateral projection one year after additional ventral augmentation showing no further loss of correction

A 46-year-old Caucasian woman underwent surgery with a Harrington intrumentation (T4-L3) due to idiopathic scoliosis 30 years ago. During the operation she received a blood transfusion and was infected with hepatits C. Today she has cirrhosis with liver failure (Child-Pugh classification C). Without any trauma she felt a sudden pain in her lower back with paraesthesis in both her legs but no other neurological symptoms. A computed tomography (CT) scan (Figure 1) and X-ray (Figures 2, 3) showed a bilateral pedicle fracture of L3. We first performed a dorsal instrumentation L1-5 and a posterolateral spondylodesis. She had massive intraoperative bleeding and a very bad quality of the bone. Due to the bad quality of the bone we were not able to place the pedicle screws in L1 bilaterally. She needed a perioperative mass transfusion of fresh-frozen plasma (FFP), PPSB and erythrocyte concentrates which is why we were not able to perform a ventral augmentation in one session as originally planed. Postoperatively the liver function decompensated and the patient needed to stay in the intensive care unit for ten days and had a notably extended rehabilitation. Although she was carefully mobilized with a corset she developed a progressive thoracolumbar kyphosis with a looming cutting-out of the pedicle screw in L1 (Figures 4, 5). We needed to perform an additional ventral augmentation T11-L3 with the same problems that occurred during the first operation with another mass transfusion of blood products. After the second operation the patient developed a hepatorenal syndrome and needed to stay in the intensive care unit for 28 days and another month in the rehabilitation unit. In summary, both operations required a long postoperative stay in the intensive care unit and led to a decompensation of her liver function. After rehabilitation, at her 12-month follow up, she is free of complaints and her X-ray shows no further loss of correction (Figures 6, 7).

## Discussion

Spontaneous fractures of the spine occur due to previous damage, be it from osteoporosis, any other systemic pathology affecting the spine or be it iatrogenic. Cyron *et al*. showed that the pedicle is the second weakest point in the neural arch after the pars interarticularis [7,8]. The pedicle remains less likely to fracture because of its shorter moment arm [9]. However, few cases of bilateral pedicle stress fractures have been reported [10-15]. Kim *et al*. described a bilateral pedicle fracture of L4 in a patient with a four-year history of ankylosing spondylitis [10]. Two cases have been reported in which no other pre-existing causative factors than osteoporosis have been found [11,12]. Tribus *et al*. described the case of a patient treated with posterior spinal fusion with segmental instrumentation from T3 to L4 who developed fractures of L4 pedicles [16]. Knight *et al*. described a fractured pedicle within the fusion mass after Harrington Instrumentation [17]. Due to the fact that these cases seldom occur, the therapy varies from conservative treatment over percutaneous instrumentation of the affected pedicles to dorsal instrumentation and spondylodesis with or without the neighboring segments. In our patient the situation was highly unstable. Even after dorsal instrumentation the patient developed a progressive kyphosis which made an additional ventral spondylodesis necessary.

## Conclusion

Bilateral pedicle fractures in the spine are very uncommon. Few cases have been reported. Many patients who underwent surgery with a Harrington Instrumentation as a child or teenager are, or will be, in an age where osteoporosis becomes an issue. Although in the case of our patient her severe liver cirrhosis made surgery even more difficult, spinal surgeons should be aware of the management of these kinds of complications. Regarding the serious complications one could retrospectively think of a less invasive surgery but to prevent further loss of correction and achieve a good long-term result some kind of bony spondylodesis is needed. This can only be achieved with open surgery which is why we would recommend a dorso-ventral procedure.

## Consent

Written informed consent was obtained from the patient for publication of this case report and any accompanying images. A copy of the written consent is available for review by the Editor-in-Chief of this journal.

## Competing interests

The authors declare that they have no competing interests.

## Authors' contributions

PO wrote the case report. AR and HÜ made substantial contributions to its conception and design. TN has given final approval of the version to be published. All authors read and approved the final manuscript.
